# Comparing the differences in three measures of healthy life expectancy among prefectures in Japan

**DOI:** 10.1186/s13104-020-05213-z

**Published:** 2020-08-05

**Authors:** Kazuya Taira, Soshiro Ogata, Kei Kamide

**Affiliations:** 1 Department of Human Health Science, Graduate School of Medicine, Kyoto University, 53, Shogoinkawara-cho, Sakyo-ku, Kyoto 606-8507 Japan; 2 Department of Preventive Medicine and Epidemiology, National Cerebral and Cardiovascular Center, Suita, Osaka Japan; 3 Faculty of Nursing, School of Health Science, Fujita Health University, Toyoake, Aichi Japan; 4 Division of Health Sciences, Graduate School of Medicine, Osaka University, Suita, Osaka Japan

**Keywords:** Healthy life expectancy, Ecological study, Subjective health, Generalized linear mixed model

## Abstract

**Objective:**

An ecological study using secondary open data from Japanese government statistics was conducted. The study aimed to verify differences in three measures of healthy life expectancy (HLE); namely, disability-free life expectancy without activity limitation (DFLE-AL), life expectancy with self-perceived health (LE-SH), and disability-free life expectancy without care need (DFLE-CN).

**Results:**

Each HLE from 47 prefectures in 2010, 2013, and 2016 was extended over time. There were strong Cronbach’s coefficient alpha (α) between DFLE-AL and LE-SH (Minimum α; 0.80, Maximum α; 0.90) as well as between LE and DFLE-CN (Minimum α; 0.92, Maximum α; 0.99) in both sexes in every data year. However, the other pairs had weaker associations. In regression analysis with each HLE as a dependent variable and aging rate, mortality, the proportion of unhealthy people as independent variables, the subjective unhealthy rate had significant standardized partial regression coefficients (β) in models with DFLE-AL and LE-SH as dependent variables (Minimum β; − 0.56, Maximum β; − 0.34). Therefore, DFLE-CN tended to differ from the other HLEs. The subjective unhealthy rate had a significant influence on DFLE-AL and LE-SH.

## Introduction

The healthy life expectancy (HLE) has been extremely important to the national health policy in Japan [1]. Three measures of HLE calculated by the Sullivan method [2] that differed according to the definition of unhealthiness have been used as target values of local government policy [3]. These HLEs, selected for this study are as follows: (i) disability-free life expectancy without activity limitation (DFLE-AL), (ii) life expectancy with self-perceived health (LE-SH), and (iii) disability-free life expectancy without care need (DFLE-CN) (Additional file 1).

Some studies [4–6] and surveys by local governments [7, 8] on factors that extend these HLEs have been reported, but these studies referred only to a single HLE, and few reports mentioned the differences between characteristics of different measures of HLE. Furthermore, even studies in which sub-concepts, such as frailty and activities of daily living, were set as objective variables instead of HLE concluded that they contributed to HLE extension. Moreover, the three measures of HLE were not distinguished and were treated as single indicators [9–12].

LE-SH and DFLE-AL can only be calculated every 3 years because the basic data for calculations are extracted from a triennial survey conducted by the Japanese government. Though Life Expectancy (LE) and HLE are consistently linearly extended in both sexes, the percentage of unhealthy periods in LE also increased [13]. Therefore, this triennial evaluation of HLE may show that its extension is influenced by LE extension. In Japanese administrative organizations, the linkage between policy and budget systems has been regarded as an issue [14]. Triennial evaluation of HLE is disadvantageous in that it cannot be linked to the project budget that is carried out every year. In policymaking, it is important to clarify the relationships between HLE, mortality, and the proportion of unhealthy people, and to identify the indicator that can be monitored annually by local governments. The single-year indicator reduces the influence of the LE extension and helps improve the projects for HLE extension every year, which in turn leads to healthy longevity of the local residents.

This study aimed to compare LE and three measures of HLE and to clarify the indicators related to each HLE that can be evaluated every year.

## Main text

### Methods

#### Study design and data resource

We conducted an ecological study using secondary open data published as government statistics for 2010, 2013, and 2016 by prefecture. The data used for analysis were LE, three measures of HLE, population by age, number of deaths, and the number of unhealthy people. Values of HLEs and LE were obtained from the “Healthy Life Report from Japan.” [3] All other variables were derived from the government statistics website, “e-Stat.” [15] The population by age was derived from the population census for 2010, and population estimates for 2013 and 2016. The number of deaths was derived from official vital statistics. There are three measures of unhealthy people for each HLE (Additional file 1). The people who answered “Yes” to the question “Do health problems currently affect your daily life in some way? (Yes/No)” for DFLE-AL, and the people who answered “Not very good” or “Not good” to the question “What is your current state of health?” on a 5-point scale rating (not good–very good) for LE-SH, were regarded as unhealthy. The data for these two questions were derived from a comprehensive survey of living conditions [16]. In DFLE-CN, people requiring long-term care (level 2 or higher) were determined to be unhealthy in a survey of long-term care benefit expenses [17]. In the analysis, these variables were converted to the following rates or ratios: aging rate, mortality, and proportion of unhealthy people.

#### Main outcome

LE and three measures of HLE were used as outcome variables.

#### Main predictors

The proportion of unhealthy people was used as the main predictor. In this paper, we defined the proportion of unhealthy people for each HLE as the restriction rate for DFLE-AL, the subjective unhealthy rate for LE-SH, and the care need rate for DFLE-CN. The restriction rate and the subjective unhealthy rate were calculated by dividing the number of unhealthy people in the self-administered questionnaire by the number of respondents in each survey. The care need rate was calculated by dividing the number of persons requiring long-term care (level 2 or higher) by the number of persons aged ≥ 40 years who were eligible for long-term care insurance.

#### Other variables

Mortality, aging rate, and data year were used as the other predictor variables. Mortality was calculated by dividing the number of deaths by the total population. Aging rate was calculated by dividing the population aged ≥ 65 years by the total population. Data year was used after converting from 1 to 3 in the order of 2010, 2013, and 2016. To assist with interpretation, all variables except data year were expressed in “per 1000 persons.”

#### Statistical analyses

First, Cronbach’s coefficient alphas (α) for LE and the three measures of HLE were calculated to confirm their similarity. Then, regression analysis was performed using a generalized linear mixed model (GLMM), in which each HLE was used as dependent variables. Data year, aging rate, mortality, and proportion of unhealthy people were included as independent variables.

**Fig. 1:**
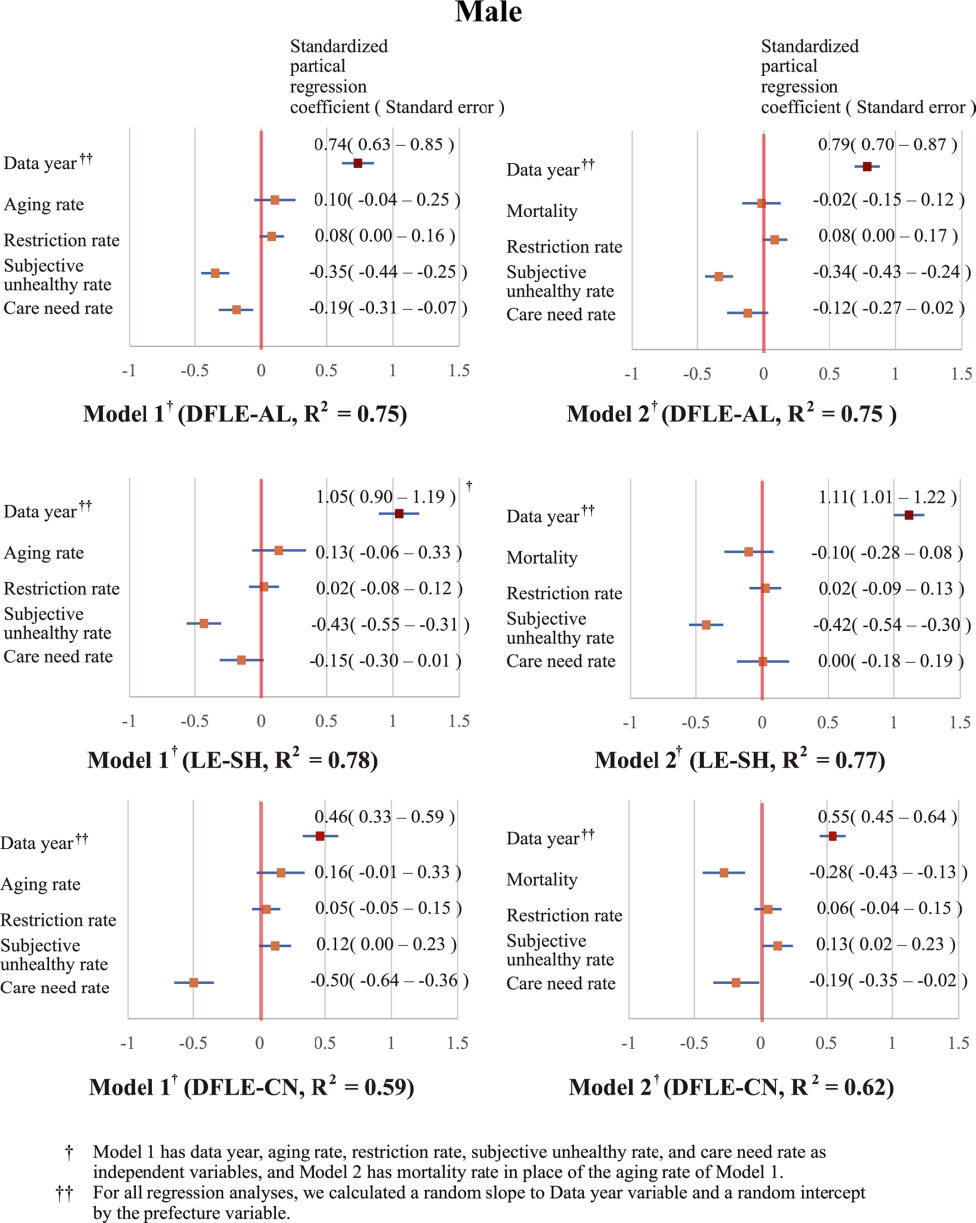
Regression analysis of the generalized linear mixed model of males with the three measures of HLE. *DFLE-AL* disability-free life expectancy without activity limitation, *DFLE-CN* disability-free life expectancy without care need, *LE* life expectancy, *LE-SH* life expectancy with self-perceived health

**Fig. 2:**
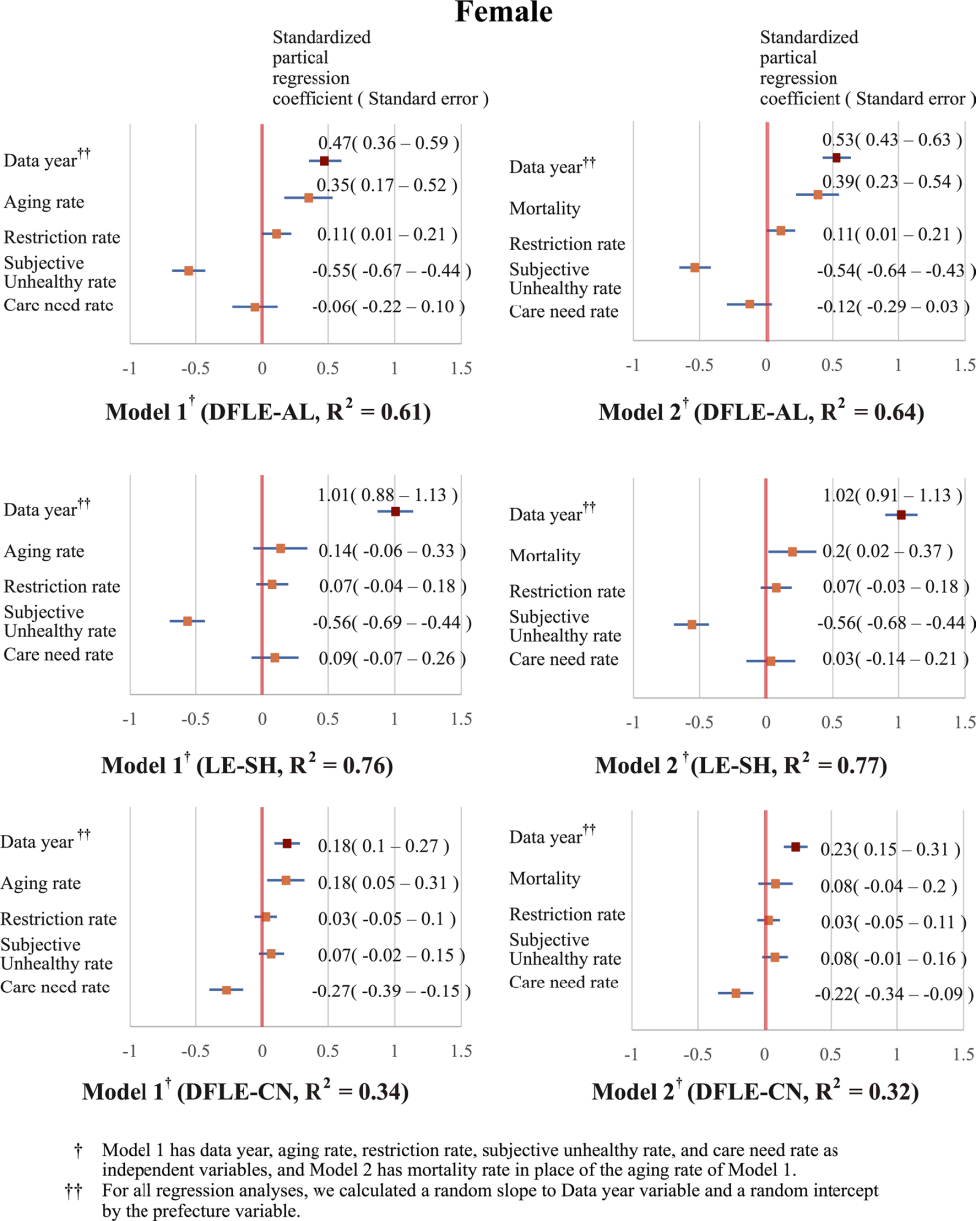
Regression analysis of the generalized linear mixed model of females with the three measures of HLE. *DFLE-AL* disability-free life expectancy without activity limitation, *DFLE-CN* disability-free life expectancy without care need, *HLE* healthy life expectancy, *LE* life expectancy, *LE-SH* life expectancy with self-perceived health

In the regression analysis, data of 47 prefectures in 2010, 2013, and 2016 were combined for all variables and treated as variables of 140 samples (the data for Kumamoto Prefecture in 2016 were missing due to an earthquake disaster). For the independent variables, the model was constructed after the evaluation of multicollinearity using a variance inflation factor (VIF). Models with both aging rate and mortality as independent variables had a high VIF value of 8.4–15.2, which could lead to multicollinearity problems. Therefore, Model 1, with data year, aging rate, restriction rate, subjective unhealthy rate, and care need rate as independent variables, and Model 2, with mortality as the independent variable in place of aging rate, were developed. In these two models, regression analyses for each HLE were performed by sex. For all regression analyses, we calculated a random slope for the data year variable and a random intercept by the prefecture variable (Figs. 1 and 2).

For estimation of the parameters, simulated draws from the posterior were obtained for each parameter using the Markov chain Monte Carlo (MCMC) method [18, 19]. The simulated draws were preceded with 2500 “burn-in” draws, which were discarded from the analysis. The MCMC chain was thinned by including only every second draw, yielding 5000 simulated posterior observations. Then, Rhat was calculated to confirm the convergence of the simulation. Rhat is an index of the divergence between chains, and in the case of three or more chains, if it is 1.1 or less by convention, it is considered to have converged.

Analyses were performed using the open source statistical software R (ver. 3.6.2) [20], and the Rstan [19] package was used for parameter estimation by MCMC.

### Results

The α between DFLE-AL and LE-SH were high for both males (Minimum α; 0.86, Maximum α; 0.90) and females (Minimum α; 0.80, Maximum α; 0.89) in all data years. However, the α between DFLE-CN and the other two measures of HLE showed moderate association in males (Minimum α; 0.61, Maximum α; 0.73) but weak association in females (Minimum α; 0.10, Maximum α; 0.48). Furthermore, the α between LE and DFLE-CN showed a strong association in both males (Minimum and Maximum α; 0.99) and females (Minimum α; 0.92, Maximum α; 0.93). However, the association between LE and the other two HLEs was moderate for males (Minimum α; 0.57, Maximum α; 0.72) and weak for females (Minimum α; 0.04, Maximum α; 0.47) (Additional file 2, Table 1).

**Table 1:** Cronbach’s coefficient alpha for life expectancy and three measures of healthy life expectancy according to year

	Male				Female			
	LE	DFLE-AL	LE-SH	DFLE-CN	LE	DFLE-AL	LE-SH	DFLE-CN
2010 (n = 47)								
LE	1.00				1.00			
DFLE-AL	0.67	1.00			0.06	1.00		
LE-SH	0.59	0.90	1.00		0.21	0.85	1.00	
DFLE-CN	0.99	0.71	0.63	1.00	0.92	0.26	0.37	1.00
2013 (n = 47)								
LE	1.00				1.00			
DFLE-AL	0.63	1.00			0.04	1.00		
LE-SH	0.70	0.86	1.00		0.25	0.89	1.00	
DFLE-CN	0.99	0.67	0.73	1.00	0.93	0.30	0.34	1.00
2016 (n = 46^a^)								
LE	1.00				1.00			
DFLE-AL	0.57	1.00			0.15	1.00		
LE-SH	0.72	0.87	1.00		0.47	0.80	1.00	
DFLE-CN	0.99	0.61	0.70	1.00	0.92	0.10	0.48	1.00

*DFLE-AL* disability-free life expectancy without activity limitation, *DFLE-CN* disability-free life expectancy without care need, *LE* life expectancy, *LE-SH* life expectancy with self-perceived health

^a^The data for Kumamoto Prefecture in 2016 are missing due to an earthquake disaster

The GLMM results for the standardized partial regression coefficients (β) in males (Fig. 1) showed that, for all models with DFLE-AL and LE-SH as dependent variables, data year (Minimum β; 0.74, Maximum β; 1.11) and subjective unhealthy rate (Minimum β; − 0.43, Maximum β; − 0.34) were significant factors. For both models with DFLE-CN as a dependent variable, the care need rate (Minimum β; -0.50, Maximum β; − 0.19) was significant.

The GLMM results in females (Fig. 2) showed that, for all models with each HLE as dependent variables, data year (Minimum β; 0.18, Maximum β; 1.02) was a significant factor. Among the models with DFLE-AL and LE-SH as dependent variables, the subjective unhealthy rate (Minimum β; − 0.56, Maximum β; − 0.54) was significant in all models. For both models with DFLE-CN as the dependent variable, the care need rate (Minimum β; − 0.22, Maximum β; − 0.27) was significant.

Finally, values of Rhat and the other indications pointed to the fact that the MCMC algorithm achieved convergence for all parameters. It was also confirmed that there was no overfitting problem by calculating the models with the minimum variables (Additional files 3 and 4).

### Discussion

The GLMM results showed that data year had the greatest influence on all three measures of HLE for both sexes. The effect of the data year was considered to be an extension of the LE due to various factors such as advances in medical technology and improvements in the living environment. This may be the cause of the extension of the unhealthy period pointed out in a previous study [13]. In addition, from the results of alpha, DFLE-AL and LE-SH had a strong association (Minimum α; 0.69, Maximum α; 0.83) for all data years, indicating that these indices are similar. Nevertheless, DFLE-CN showed a strong association (Minimum α; 0.75, Maximum α; 0.98) with LE rather than with the other two HLEs; therefore, DFLE-CN appeared to be an indicator more closely aligned to LE than the other two measures of HLE.

In DFLE-CN, a third party judges the state of health based on physical condition, which depends on the nursing care insurance system, and therefore physical aspects are strongly reflected. The other two HLEs are questionnaire-based judgments of unhealthiness, but no correlation is seen in women because they judge health in other aspects, such as psychological and social aspects. It has been reported that women tend to experience worse subjective health than men, and gender gaps in subjective health are not systematically related to socioeconomic gender inequalities [21, 22]. Hence, it is unclear as to why there is a gender gap in the subjective health (i.e., the subjective unhealthy rate).

Furthermore, the GLMM result shows that the subjective unhealthy rate influenced the DFLE-AL and LE-SH, both of which used subjective health scales involving a self-administered questionnaire. This finding supports previous studies which reported that subjective health was related to reducing mortality and incidence of cardiovascular diseases [23–26]. Other studies that examined factors which extend HLE also reported that eliminating malignant neoplasms and cerebrovascular diseases extends DFLE-CN [4] and that lifestyle habits, such as smoking cessation and vegetable intake, are associated with the extension of DFLE-CN [7, 8]. Therefore, the subjective unhealthy rate may be an indicator for predicting the extension of DFLE-AL and LE-SH as a representative measure of these factors.

From these results, it can be said that it is necessary to evaluate the extension of DFLE-CN in consideration of its strong correlation to the extension of LE and that the subjective unhealthy rate can be useful as a single-year indicator for evaluating LE-SH and DFLE-AL. Annual evaluation of the indicator enables local governments to evaluate and improve projects for extending HLE while reducing the influence of LE extension. This will lead to a long and healthy life for local residents.

### Conclusion

Over time, all three HLEs were more correlated with increased LE. DFLE-AL and LE-SH are strongly related, and the evaluation excluding the effect of LE is necessary for DFLE-AL. The subjective unhealthy rate had a significant influence on prefectural DFLE-AL and LE-SH.

## Limitations

This ecological study was conducted on a prefectural basis and could not assess HLE at an individual level. Further, the care need rate was calculated by dividing the population at age ≥ 40 years; however, the rate may have been underestimated due to the large denominator, as people aged between 40 and 65 years and eligible for long-term care insurance are limited to those with certain diseases.

## Supplementary information

**Additional file 1.** The definition of three measures of healthy life expectancy in Japan.

**Additional file 2.** Descriptive statistics according to sex.

**Additional file 3.** Regression analysis of the generalized linear mixed model of males with the three measures of HLE and minimal independent variables.

**Additional file 4.** Regression analysis of the generalized linear mixed model of females with the three measures of HLE and minimal independent variables.

## Data Availability

The datasets generated and/or analysed during the current study are available in the portal website of official statistics of Japan “e-Stat”, https://www.e-stat.go.jp/en/, and the websites “Healthy Life Reports from Japan”, http://life.umin.jp/.

## Abbreviations

LE: Life expectancy
HLE: Healthy life expectancy
DFLE-AL: Disability-free life expectancy without activity limitation
LE-SH: Life expectancy with self-perceived health
DFLE-CN: Disability-free life expectancy without care need
ADL: Activities of daily living
IADL: Instrumental activities of daily living
GLMM: Generalized linear mixed model
MCMC: Markov chain Monte Carlo method
α: Cronbach’s coefficient alpha
β: Standardized partial regression coefficients
