# Supplementary material for: Comparing the differences in three measures of healthy life expectancy among prefectures in Japan
Source: BMC Res Notes. 2020 Aug 5;13:371. doi: 10.1186/s13104-020-05213-z (PMC7404923; doi:10.1186/s13104-020-05213-z)
Supplement: Supplementary file 1 — Additional file 1. The definition of three measures of healthy life expectancy in Japan. [file 13104_2020_5213_MOESM1_ESM.docx]

**Additional file.1** The definition of three measures of healthy life expectancy in Japan

|  | Healthy Life Expectancy | Definition of Healthy/Unhealthy |
| --- | --- | --- |
| Japan | Disability-free life expectancy without activity limitation  (DFLE-AL) | Do health problems currently affect your daily life in some way? (Yes/No) |
|  | Life expectancy with self-perceived health (LE-SH) | How is your health at present? Circle the response that applies to you.  (Good/ Fairly good/Ordinary/Not very good/Not good) |
|  | Disability-free life expectancy without care need (DFLE-CN) | Care need levels in the long-term care insurance system (levels 1–3/levels 4–7) |
