# Supplementary material for: Comparing the differences in three measures of healthy life expectancy among prefectures in Japan
Source: BMC Res Notes. 2020 Aug 5;13:371. doi: 10.1186/s13104-020-05213-z (PMC7404923; doi:10.1186/s13104-020-05213-z)
Supplement: Supplementary file 2 — Additional file 2. Descriptive statistics according to sex. [file 13104_2020_5213_MOESM2_ESM.docx]

**Additional file.2** Descriptive statistics according to sex

|  | n | Male | Female |
| --- | --- | --- | --- |
|  |  | Mean ± SD | Mean ± SD |
| DFLE-AL (2010–2016) | 140 | 71.2 ± 0.9 | 74.4 ± 0.9 |
| 2010 | 47 | 70.42 ± 0.68 | 73.75 ± 0.77 |
| 2013 | 47 | 71.21 ± 0.59 | 74.39 ± 0.72 |
| 2016 | 46 | 72.05 ± 0.51 | 74.94 ± 0.66 |
| LE-SH (2010–2016) | 140 | 71.1 ± 1.2 | 74.7 ± 1.2 |
| 2010 | 47 | 69.89 ± 0.81 | 73.45 ± 0.78 |
| 2013 | 47 | 71.24 ± 0.70 | 74.92 ± 0.68 |
| 2016 | 46 | 72.23 ± 0.66 | 75.72 ± 0.72 |
| DFLE-CN (2010–2016) | 140 | 78.7 ± 0.8 | 83.5 ± 0.5 |
| 2010 | 47 | 78.09 ± 0.63 | 83.22 ± 0.43 |
| 2013 | 47 | 78.61 ± 0.64 | 83.41 ± 0.43 |
| 2016 | 46 | 79.34 ± 0.62 | 83.81 ± 0.43 |
| LE (2010–2016) | 140 | 80.15 ± 0.82 | 86.71 ± 0.51 |
| 2010 | 47 | 79.56 ± 0.81 | 86.43 ± 0.78 |
| 2013 | 47 | 80.09 ± 0.70 | 86.63 ± 0.68 |
| 2016 | 46 | 80.82 ± 0.66 | 87.08 ± 0.72 |
| Aging rate (per 1000 population)^†^ | 140 | 234.2 ± 28.9 | 296.3 ± 36.5 |
| Death rate (per 1000 population)^†^ | 140 | 11.6 ± 1.7 | 10.3 ± 1.7 |
| ^†^Restriction rate  (per 1000 population) | 140 | 375.1 ± 21.3 | 412.0 ± 23.4 |
| SH rate (per 1000 population)^†^ | 140 | 130.2 ± 10.7 | 130.2 ± 10.7 |
| CN rate (per 1000 population)^†^ | 140 | 30.9 ± 4.2 | 58.7 ± 8.4 |

^†^Average and standard deviation of 2010, 2013 and 2016 data for all 47 prefectures

**Abbreviations:** CN, care need; DFLE-AL, disability-free life expectancy without activity limitation; DFLE-CN, disability-free life expectancy without care need; LE, life expectancy; LE-SH, life expectancy with self-perceived health; SD, standard deviation; SH, self-perceived health
