# Supplementary material for: Comparing the differences in three measures of healthy life expectancy among prefectures in Japan
Source: BMC Res Notes. 2020 Aug 5;13:371. doi: 10.1186/s13104-020-05213-z (PMC7404923; doi:10.1186/s13104-020-05213-z)
Supplement: Supplementary file 4 — Additional file 4. Regression analysis of the generalized linear mixed model of females with the three measures of HLE and minimal independent variables. [file 13104_2020_5213_MOESM4_ESM.docx]

**
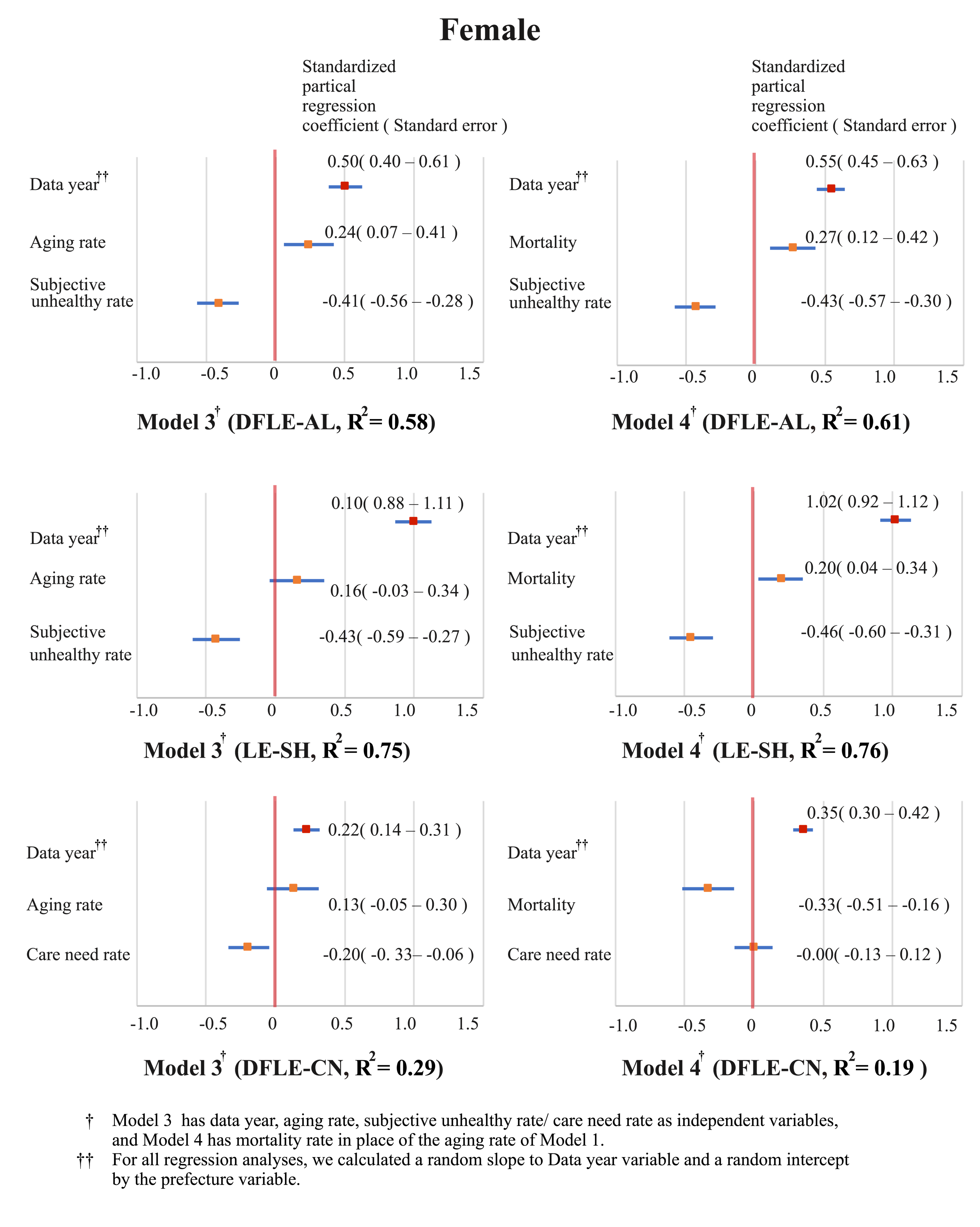
**

**Additional file.4** Regression analysis of the generalized linear mixed model of females with the three measures of HLE and minimal independent variables
